# Dietary Protein Restriction Ameliorates Cardiac Inflammaging via AMPK‐ULK1‐Mediated Mitochondrial Quality Control

**DOI:** 10.1111/acel.70386

**Published:** 2026-01-18

**Authors:** Wagner S. Dantas, Elizabeth R. M. Zunica, Elizabeth C. Heintz, Charles L. Hoppel, Cristal M. Hill, Christopher D. Morrison, Christopher L. Axelrod, Gangarao Davuluri, John P. Kirwan

**Affiliations:** ^1^ Integrated Physiology and Molecular Medicine Laboratory, Pennington Biomedical Research Center Louisiana State University Baton Rouge Louisiana USA; ^2^ Department of Human Physiology University of Oregon Eugene Oregon USA; ^3^ Department of Pharmacology Case Western Reserve University Cleveland Ohio USA; ^4^ Leonard Davis School of Gerontology University of Southern California Los Angeles California USA; ^5^ Neurosignaling Laboratory, Pennington Biomedical Research Center Louisiana State University Baton Rouge Louisiana USA; ^6^ Responsive Agricultural Food Systems Research Unit USDA‐ARS College Station Texas USA

**Keywords:** bioenergetics, fission, fusion, heart, mitochondria, obesity, quality control

## Abstract

Calorie restriction (CR) is a robust intervention for improving metabolic health and delaying obesity and age‐related diseases, yet its translational utility is limited by adherence challenges and diminished effectiveness later in life. Dietary protein restriction (DPR), which reduces dietary protein without decreasing total caloric intake, has emerged as a promising alternative, yet its cardioprotective potential in the context of obesity and aging remains poorly understood. Here, we demonstrate that DPR mitigates obesity‐induced cardiac remodeling and inflammaging by activating the AMPK–ULK1 signaling axis and enhancing mitochondrial quality control. In middle‐aged male mice with high‐fat diet‐induced obesity, 4 months of DPR attenuated cardiac hypertrophy and normalized heart failure markers, independently of FGF21 signaling. Transcriptomic and protein analyses revealed that DPR suppressed the activation of the cGAS–STING pathway, reduced mitochondrial DNA release into the cytosol, and blunted expression of pro‐inflammatory mediators, including IRF3 and IFN‐γ. DPR also restored mitochondrial dynamics, enhanced mitophagy, and maintained ATP content despite reduced respiratory capacity. Mechanistically, DPR increased AMPK‐dependent ULK1 phosphorylation while suppressing mTOR signaling, thereby promoting mitochondrial turnover. These effects were confirmed in cardiomyocytes, where AMPK knockdown abrogated ULK1 activation and mitophagy under conditions of low amino acid availability. Together, these findings uncover a novel mechanism by which DPR attenuates cardiac inflammation and supports mitochondrial homeostasis, highlighting its therapeutic potential for enhancing cardiovascular health during obesity‐mediated inflammaging.

## Graphical abstract

Dietary protein restriction protects the aging heart in the context of obesity by limiting mitochondrial DNA leakage and suppressing cGAS–STING‐driven inflammation. Through AMPK–ULK1‐dependent mitophagy, protein restriction restores mitochondrial quality control, reduces cardiac remodeling, and preserves metabolic homeostasis during obesity‐associated aging.

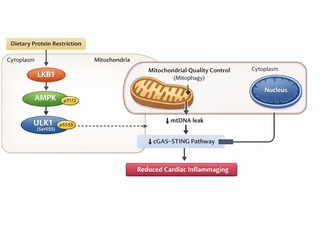

## 1 Introduction

A central focus of nutritional research features the cellular and molecular impact of dietary influence on health span. A range of nutritional interventions, including calorie restriction (CR), intermittent fasting, fasting mimetics, and dietary restriction, have been shown to improve metabolic health and extend lifespan (Fontana and Partridge 2015). Although the precise role of reduced dietary macronutrients in mediating the benefits of CR remains under investigation, there is growing evidence that macronutrient balance exerts a profound influence on longevity and overall health (Simpson and Raubenheimer 2009).

The global population is expected to live on average for 77.3 years by 2050, up 4.5 years since 2021 (GBD 2021 Forecasting Collaborators 2024). In addition, the sustained increase in obesity prevalence over recent decades is well documented (Kachmar et al. 2025). The extension of lifespan despite rising obesity rates highlights the urgent need to identify and evaluate effective geroprotective strategies that can prevent or delay the onset of both obesity‐related and age‐associated diseases. Among the most extensively studied and consistently effective interventions is CR, which typically involves a 20%–40% reduction in caloric intake relative to ad libitum feeding (Green et al. 2022). Although CR robustly reduces the incidence of numerous obesity and age‐associated diseases, long‐term adherence is difficult to maintain without risking malnutrition (Mihaylova et al. 2023). Moreover, the efficacy of CR diminishes when initiated later in life (Hahn et al. 2019), raising concerns about its feasibility and effectiveness as a widespread public health strategy.

High‐protein diets have long been advocated for weight loss and the prevention of obesity and its metabolic consequences (Leidy et al. 2015). Popularized in the 1960s, these diets remain a cornerstone of weight management. While their long‐term impact on cardiovascular health remains unclear, high‐protein diets have been assumed to confer protective effects against cardiovascular disease by improving body composition and metabolic risk profiles (Song et al. 2016). However, emerging epidemiological evidence suggests an inverse relationship: lower protein intake is associated with improved metabolic health and resilience, whereas higher protein consumption is associated with increased mortality (Levine et al. 2014). Supporting this, studies in rodent models (Zhang et al. 2020) and nonhuman primates (Cox et al. 2017) have linked high dietary protein intake to the development and progression of cardiovascular disease. Despite these findings, direct mechanistic studies exploring how high protein intake contributes to cardiovascular pathology are limited, and the underlying biological pathways remain poorly defined.

In contrast, dietary protein restriction (DPR)—a strategy that reduces protein intake without lowering total caloric intake—has been shown to extend lifespan and enhance metabolic health across several species (Solon‐Biet et al. 2014). DPR uniquely extends lifespan, whereas fat or carbohydrate restriction does not yield similar benefits (Lee et al. 2008), indicating that the longevity benefits of DPR are independent of caloric intake (Hill et al. 2022). Moreover, restricting specific amino acids such as methionine, threonine, tryptophan, or branched‐chain amino acids (BCAAs) also promotes lifespan extension, suggesting that DPR activates distinct molecular pathways that support metabolic health and longevity, even under conditions of obesity (Hill et al. 2022). However, whether DPR can improve cardiovascular outcomes during aging in the context of obesity and the molecular mechanisms involved remain unclear.

In this study, we demonstrate that dietary DPR attenuates cardiac inflammaging, characterized by chronic, low‐grade inflammation in aged hearts with obesity, through the activation of the AMPK‐UNC51‐like kinase 1 (ULK1) signaling axis, thereby promoting mitochondrial quality control. These findings provide novel mechanistic insight into how reduced dietary protein intake exerts cardioprotective effects, underscoring the potential of DPR as a strategy for promoting cardiovascular health during aging with obesity.

## 2 Results

### 2.1 Dietary Protein Restriction Mitigates Cardiac Remodeling During Obesity in Middle‐Aged Mice

**FIGURE 1:**
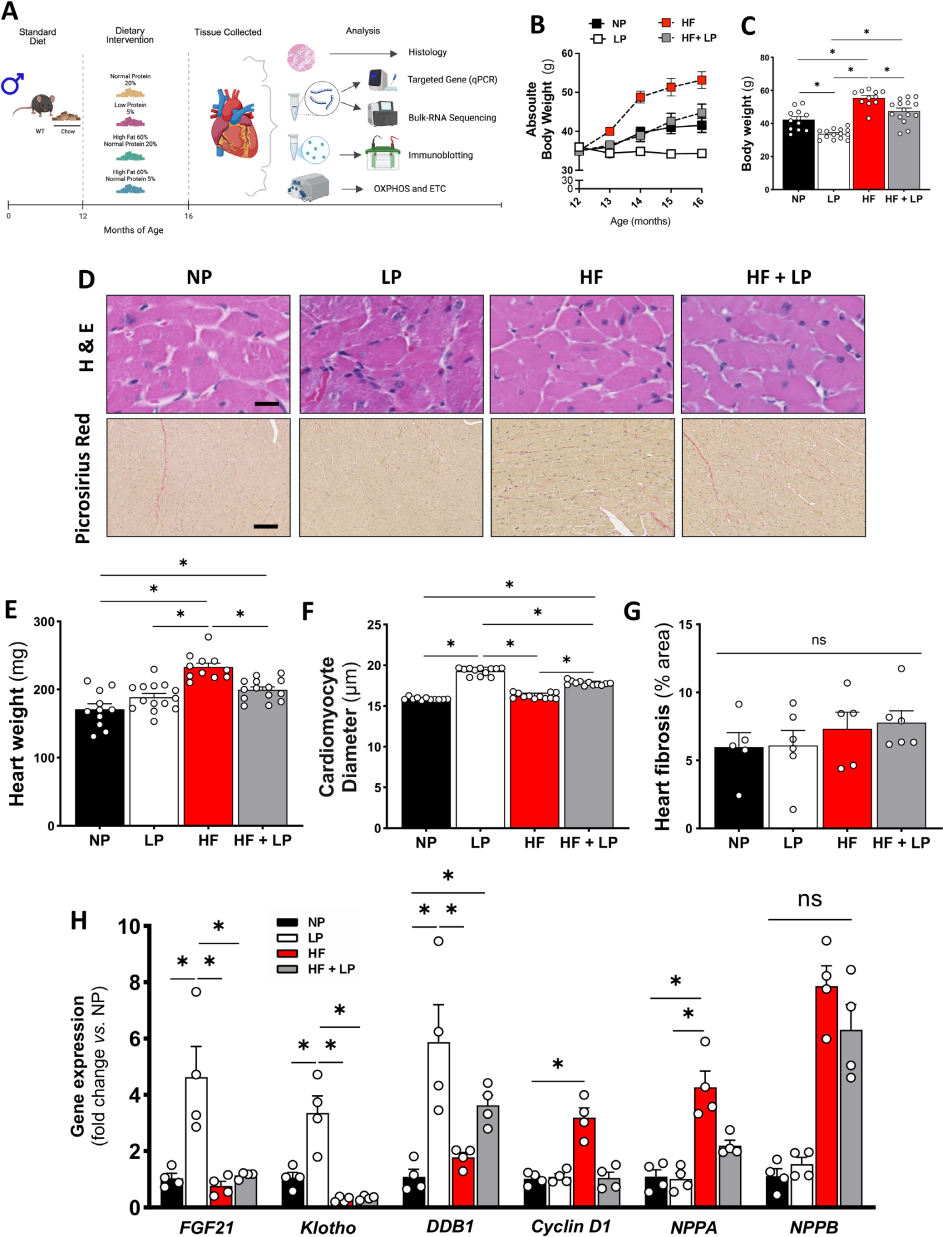
Effects of dietary protein restriction and high‐fat diet on cardiac remodeling. (A) Experimental design. (B) Absolute body weight over time. (C) Body weight at 16 months of age. (D) Representative images of heart sections stained with hematoxylin and eosin (H&E) and Picrosirius Red (scale bars in black = 50 μm). (E) Heart weight. (F) Quantification of cardiomyocyte diameter (*n* = 10–15 per group) and (G) cardiac fibrosis (*n* = 5 per group). (H) Gene expression in response to different dietary interventions (*n* = 4 per group). Normal protein (NP), low protein (LP), high‐fat (HF), and high‐fat plus low protein (HF + LP). Data are shown as the mean ± SEM. * indicates *p* < 0.05 for between‐group comparison.

Hill et al. (2022) reported that dietary protein restriction induces metabolic benefits in middle‐aged mice with diet‐induced obesity. Diet intervention was initiated at 12–16 months of age. At 16 months of age, the low protein (LP) diet significantly reduced body weight compared to the normal protein (NP) diet (Figure 1C). The protein leverage theory proposes that protein intake is prioritized over other macronutrients in regulating dietary behavior (Simpson and Raubenheimer 2005). When the proportion of protein in the diet is low, organisms compensate by increasing total food intake to achieve a target protein intake (Hill and Morrison 2019). However, DPR induces metabolic stress that triggers a neuroendocrine adaptation increasing energy expenditure through a fibroblast growth factor 21 (FGF21)‐dependent mechanism, independent of food intake (Laeger et al. 2016; Hill et al. 2017). As expected, the high‐fat (HF) diet markedly increased body weight compared to all other groups, while the high‐fat with low protein (HF + LP) diet attenuated weight gain compared to the HF group (Figure 1C). Changes in body weight and composition following dietary protein restriction were attributed to enhanced energy expenditure and glucose tolerance, accompanied by lower fasting insulin levels, independent of changes in food intake (Hill et al. 2022). This ultimately reduces the lipogenic effects of hyperinsulinemia associated with obesity to promote fat gain (Tricò et al. 2024).

Here, we assess the impact of dietary protein restriction on the heart of middle‐aged male mice. Heart weight was significantly increased in the HF group, and this effect was mitigated by DPR (Figure 1E). Cardiomyocyte diameter was elevated in both protein‐restricted groups (Figure 1D,F), with no evidence of cardiac fibrosis (Figure 1D,G). Notably, heart failure markers such as *Cyclin D1* and atrial natriuretic peptide (*NPPA*) were upregulated in the hearts of obese mice and normalized in the HF + LP group (Figure 1H). LP significantly increased *DDB1* expression, a marker of DNA repair, while HF + LP suppressed it (Figure 1H). Although fibroblast growth factor 21 (*FGF21*) and its co‐receptor, *Klotho*, are implicated in mediating DPR benefits, diet‐induced obesity by HF appears to blunt their response (Figure 1H). In summary, DPR attenuates cardiac remodeling during obesity.

### 2.2 Dietary Protein Restriction Protects Against Mitochondrial DNA Leak and Cardiac Inflammaging

**FIGURE 2:**
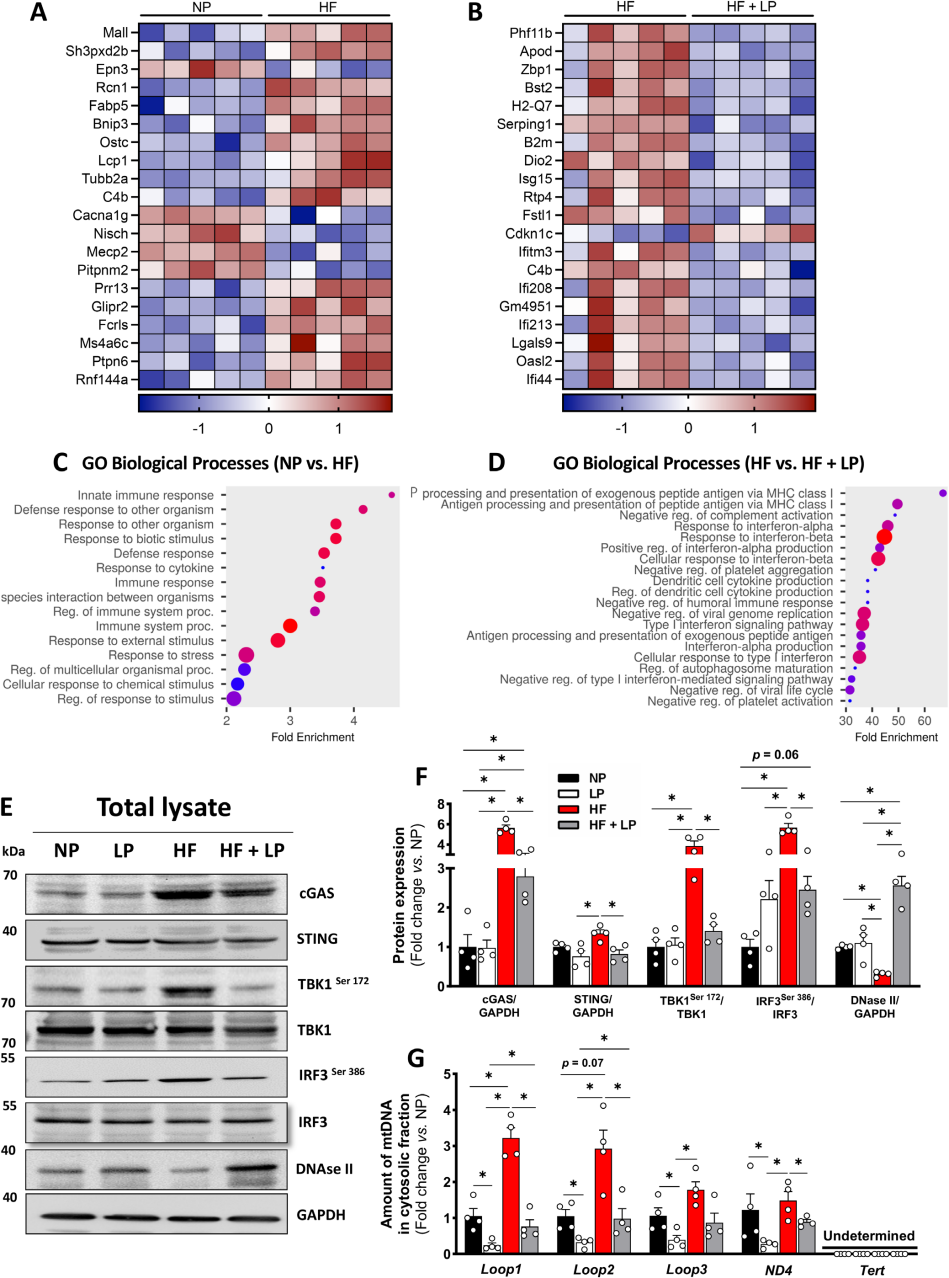
Transcriptomics changes in cardiac tissue following a high‐fat diet and dietary protein‐restricted conditions. (A, B) Heat maps visualizing the top 20 differentially expressed genes of normal dietary protein versus high‐fat diet (left) and high‐fat versus high‐fat diet combined with dietary protein restriction (right) (*n* = 6 per group). (C, D) Canonical biological pathway enrichment comparison between normal dietary protein versus high‐fat diet (left) and high‐fat versus high‐fat diet combined with dietary protein restriction (right). (E–F) Representative immunoblots and densitometric quantification of cGAS, STRING, phosphorylated and total TBK1 (Ser172), IRF3 (Ser386), DNAse II, and GAPDH (loading control) in total heart lysates (*n* = 4 per group). (G) Relative gene expression of mitochondrial DNA in the cytosolic fraction in total heart samples (*n* = 4 per group). Normal protein (NP), low protein (LP), high‐fat (HF), and high‐fat plus low protein (HF + LP). Data are shown as the mean ± SEM. * indicates *p* < 0.05 for between‐group comparison.

We conducted an unbiased transcriptomic analysis of left ventricular heart tissue from mice fed either NP, LP, HF, and HF + LP to identify the molecular pathways through which DPR attenuates cardiac remodeling during aging and obesity. Compared to NP, HF was associated with widespread activation of cardiac immune response and stress‐sensing pathways (Figure 2A,C). In contrast, HF + LP was associated with the reversal of inflammatory immune activation pathways compared to HF (Figure 2B,D). It is well‐established that activation of the cGAS–STING pathway promotes cardiac inflammation by sensing cytosolic mitochondrial DNA and triggering downstream type I interferon and pro‐inflammatory cytokine responses (Bai et al. 2017). We further confirmed at the protein level that obesity activates the cGAS‐STING pathway and its downstream targets, whereas DPR suppresses this activation in the hearts of obese mice (Figure 2E,F). Notably, deoxyribonuclease II (DNAse II) protein was reduced in the HF group but significantly increased by DPR during obesity (Figure 2E,F).

To determine whether nuclear or mitochondrial DNA contributes to activation of the cGAS‐STING pathway in the heart, we isolated and purified total DNA from the cytosolic fraction and performed RT‐PCR analysis for specific markers of nuclear and mitochondrial DNA. Expression of the nuclear marker *Tert1* was not detected in the cytosolic fraction, indicating the absence of nuclear DNA (Figure 2G). In contrast, several mitochondrial DNA markers were significantly elevated in the cytosol following the HF diet, suggesting mitochondrial DNA leakage into the cytoplasm. Notably, this effect was attenuated in the HF + LP group, which exhibited reduced expression of mitochondrial DNA markers in the cytosolic fraction (Figure 2G), suggesting that dietary DPR limits the mitochondrial DNA release and, consequently, reduces activation of the cGAS‐STING pathway.

**FIGURE 3:**
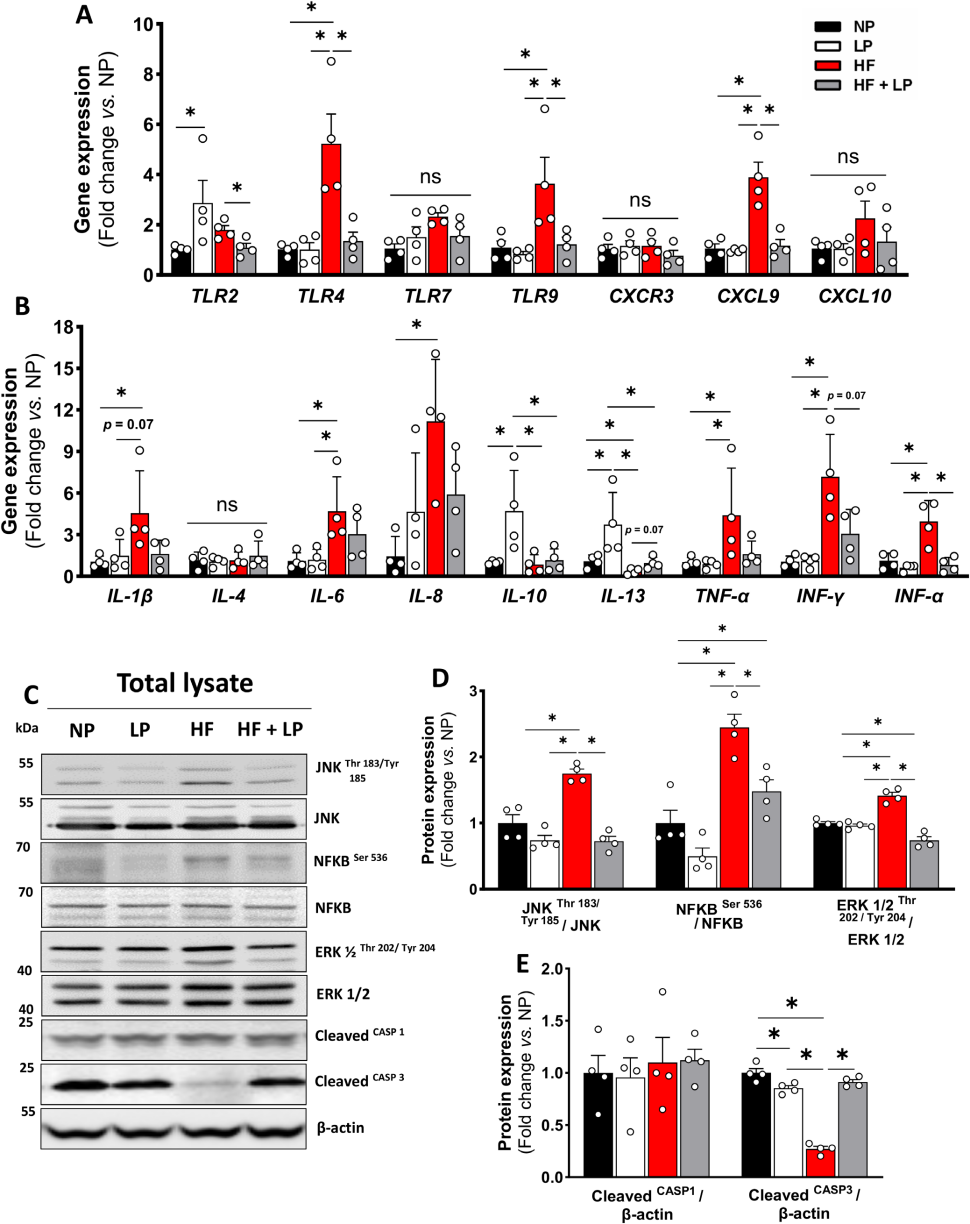
Activation of the cGAS–STING signaling under high‐fat diet and dietary protein‐restricted conditions. (A) Relative gene expression markers of toll‐like receptors (*TLR*), C‐X‐C chemokine receptor (*CXCR*), and C‐X‐C chemokine ligand (*CXCL*) in heart tissue (*n* = 4 per group). (B) Relative gene expression markers of interleukin (IL), tumor necrosis factor‐alpha (*TNF‐α*), and interferon (*INF*) in heart tissue (*n* = 4 per group). (C, E) Representative immunoblots and densitometric quantification of phosphorylated and total JNK (Thr183/Tyr185), NF‐κB (Ser536), ERK1/2 (Thr202/Tyr204), and GAPDH (loading control) in total heart lysates (*n* = 4 per group). (D, F) Representative immunoblots and densitometric quantification of cleaved caspase 1, cleaved caspase 3, and β‐actin (loading control) in total heart lysates (*n* = 4 per group). Normal protein (NP), low protein (LP), high‐fat (HF), and high‐fat plus low protein (HF + LP). Data are shown as the mean ± SEM. * indicates *p* < 0.05 for between‐group comparison.

To determine whether reduced mitochondrial DNA release into the cytosol is associated with decreased inflammation, we analyzed the expression of several innate immune activation genes in heart tissue across the different diet groups. Toll‐like receptors 4 and 9 (*TLR4* and *TLR9*, respectively) were significantly upregulated following the HF diet, but this effect was suppressed entirely in the HF + LP group (Figure 3A). Although *TLR2* expression was upregulated in the hearts of mice fed the low‐protein (LP) diet, previous studies have shown that moderate TLR2 activation can mediate protective adaptations under stress by limiting maladaptive remodeling (Spurthi et al. 2018). Thus, the role of TLR2 in cardiac physiology appears to be context‐dependent, with either protective or detrimental effects (Zhang, Wu, et al. 2023). While no significant differences were observed in chemokine receptor 3 (*CXCR3*) or interferon gamma‐induced protein 10 (*CXCL10*) expression, the chemokine ligand 9 (*CXCL9*) was markedly elevated in the HF group and normalized by the HF + LP diet (Figure 3A). Although classical pro‐inflammatory cytokines—including interleukin‐1β, 6, and 8 (*IL‐1β*, *IL‐6*, and *IL‐8*, respectively) and tumor necrosis factor‐alpha (*TNF‐α*)—were elevated in the heart following the HF diet, DPR did reduce their expression (Figure 3B). Similarly, *IL‐10* expression, an anti‐inflammatory cytokine, was suppressed by obesity, while *IL‐4* levels remained unchanged across groups (Figure 3B). In contrast, interferon‐gamma (*IFN‐γ*) and interferon‐alpha (*IFN‐α*), key mediators in the immune response to damage‐associated molecular patterns (DAMPs) (Ma et al. 2024), were significantly increased by the HF diet and normalized in the HF + LP group (Figure 3B). Consistently, activation of protein kinases involved in DAMP signaling—c‐Jun N‐terminal kinase (JNK), nuclear factor‐κB (NFKB), and extracellular signal‐regulated kinase (ERK) (Kono and Rock 2008)—was elevated with the HF diet and entirely suppressed by DPR during obese conditions (Figure 3C,D).

These molecular changes in cardiac tissue raise essential questions regarding cell fate decisions under dietary protein restriction in the context of obesity. Given that mitochondrial DNA (mtDNA) leakage and subsequent activation of cGAS–STING signaling can engage inflammatory and cell death pathways, we examined markers of pyroptosis and apoptosis. Cleaved caspase‐1 serves as a readout of inflammasome activation and cardiac inflammaging, whereas cleaved caspase‐3 reflects executioner apoptosis in cardiomyocytes (Zhou et al. 2011). Obesity reduced caspase‐3 activation without altering caspase‐1 activation (Figure 3C,E), consistent with impaired apoptotic quality control rather than inflammasome‐driven pyroptotic cell death, which is characterized by membrane rupture, IL‐1β release, and chronic inflammation (Whelan et al. 2010). In contrast, DPR under obese conditions restored caspase‐3 cleavage, suggesting reengagement of regulated apoptosis that limits the accumulation and release of DAMPs, including mtDNA, that exacerbate cardiac inflammaging (Bock and Tait 2020). Collectively, these findings support the concept that DPR shifts cardiac cell fate toward controlled apoptotic quality control while suppressing maladaptive innate immune activation, thereby mitigating obesity‐associated cardiac inflammaging.

### 2.3 Dietary Protein Restriction Restores Mitochondrial Dynamics and Quality Control During Aging With Obesity

**FIGURE 4:**
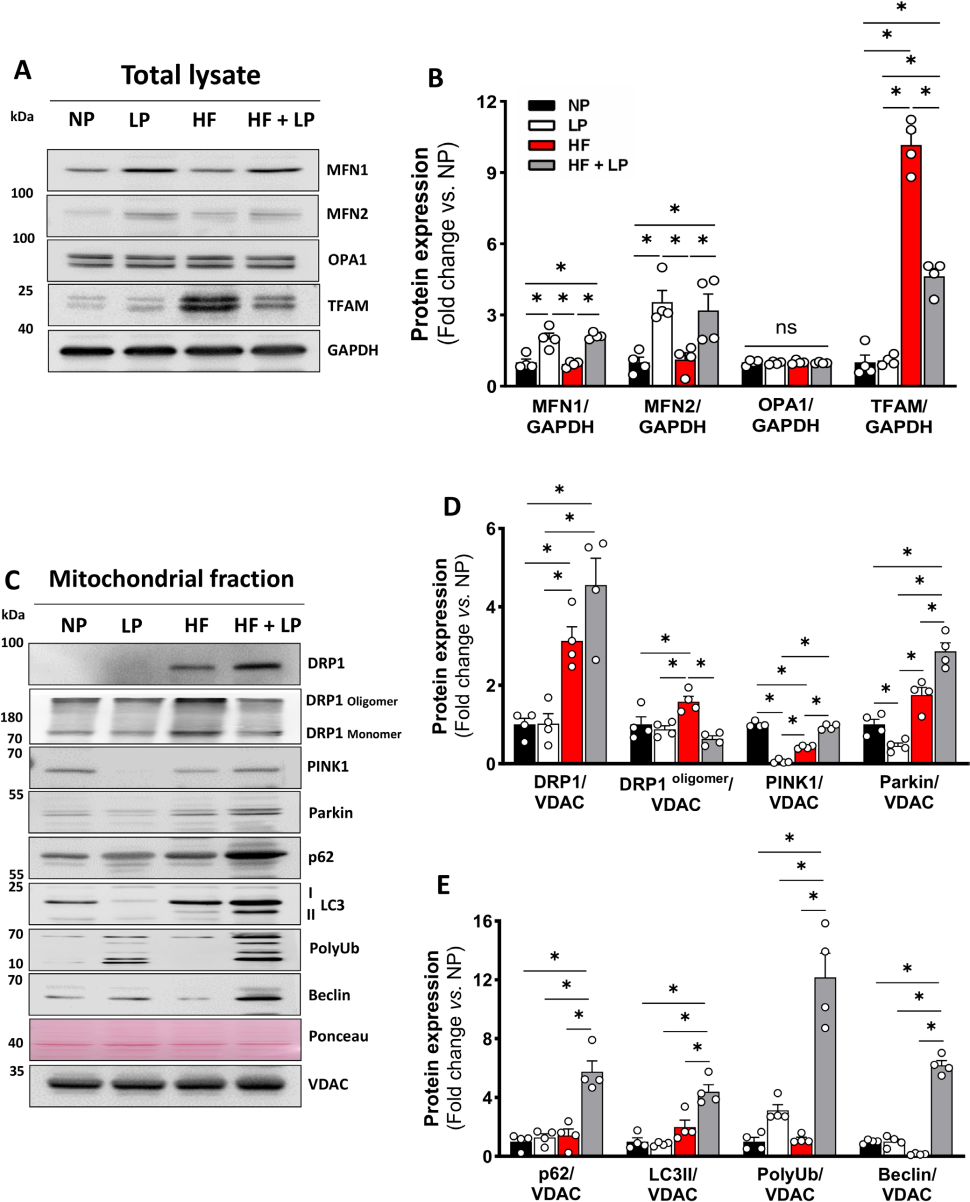
Impact of dietary protein restriction on mitochondrial dynamics and mitophagy. (A, B) Representative immunoblots and densitometric quantification of mitochondrial dynamics markers and GAPDH (loading control) in total heart lysates (*n* = 4 per group). (C–E) Representative immunoblots and densitometric quantification of mitochondrial dynamics and mitophagy markers in isolated mitochondria (*n* = 4 per group). Normal protein (NP), low protein (LP), high‐fat (HF), and high‐fat plus low protein (HF + LP). Data are shown as the mean ± SEM. * indicates *p* < 0.05 for between‐group comparison.

DPR protects mitochondria, as evidenced by reduced mitochondrial DNA leakage into the cytosol following obesity (Figure 3C). To explore the molecular mechanisms underlying this beneficial effect, we assessed the expression of key proteins involved in mitochondrial dynamics and quality control. As part of the mitochondrial fusion machinery, mitofusin 1 and 2 (MFN1 and MFN2, respectively) were significantly increased by DPR, irrespective of obesity (Figure 4A,B), whereas optic atrophy 1 (OPA1) expression was unchanged (Figure 4A,B). The expression of mitochondrial transcription factor A (TFAM), a protein that enhances both transcription and replication of mtDNA (Lee et al. 2014) was remarkably increased in the HF but significantly reduced in the HF + LP group (Figure 4A,B). While total DRP1 expression was elevated in isolated mitochondria for both obese groups, only the HF diet significantly increased DRP1 oligomerization (Figure 4C,D), indicating hyperactivation of the fission machinery during aging with obesity.

**FIGURE 5:**
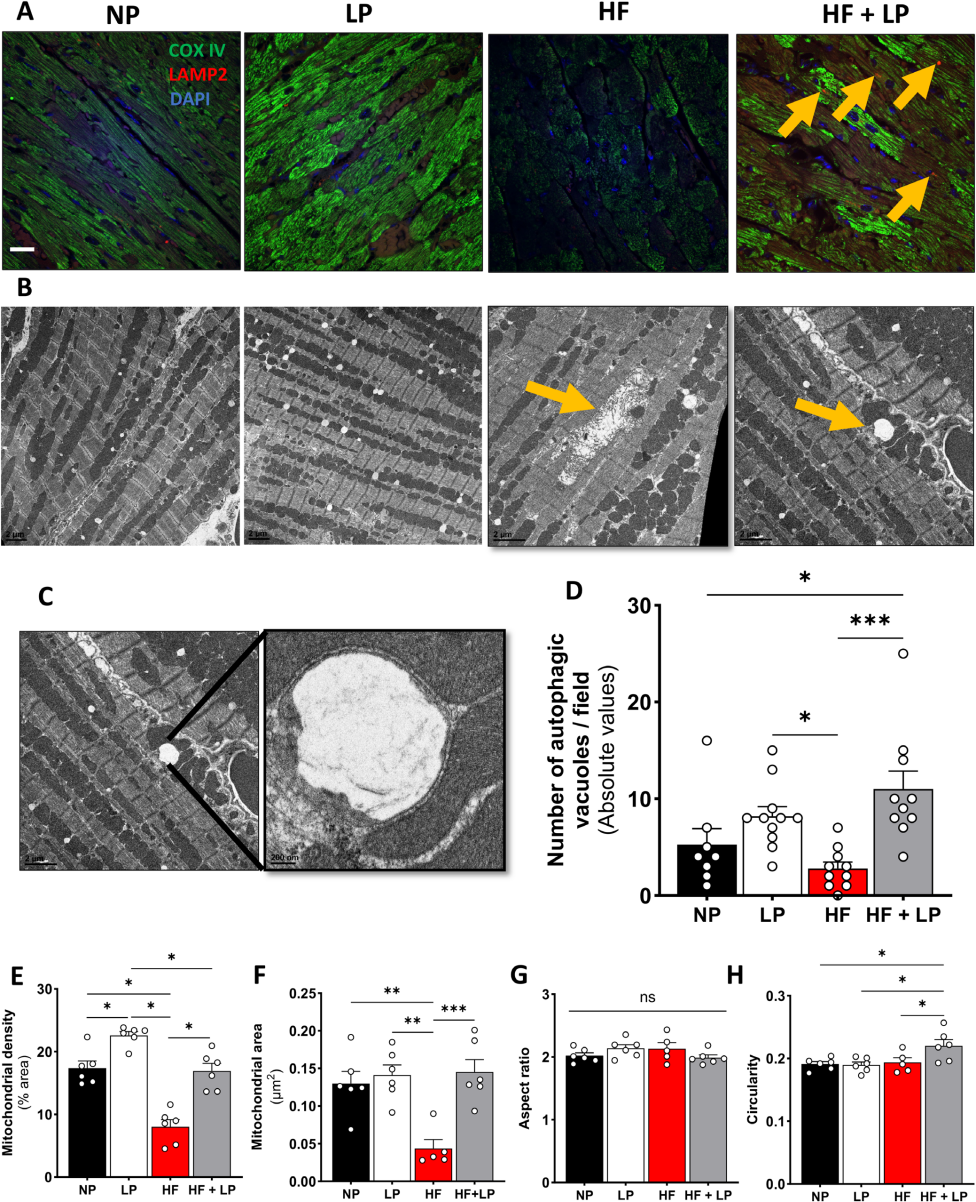
Expression of proteins regulating mitochondrial quality control. (A) Representative images of heart left ventricle sections showed positive co‐staining for lysosome‐associated membrane protein 2 (LAMP2; red dots and yellow arrows) and cytochrome c oxidase IV (COX IV; green) (scale bars in white = 10 μm). (B) Representative images of mitochondrial intermyofibrillar content from transmission electron micrographs (scale bars (black) = 2 μm). Yellow arrows indicate the presence of damaged mitochondria in the high‐fat (HF) diet group and the presence of autophagosomes surrounding mitochondria in the high‐fat plus low protein (HF + LP) group. (C, D) Representative image and quantification of autophagosomes surrounding mitochondria from transmission electron micrographs (scale bars in black = 0.5 μm) (*n* = 8–10 per group, two different one‐micrometer micrograph fields analyzed per mouse). (E–H) Mitochondrial density, mitochondrial area, aspect ratio, and circularity (*n* = 6–7 per group). Normal protein (NP), low protein (LP), high‐fat (HF), and high‐fat plus low protein (HF + LP). Data are shown as the mean ± SEM. * indicates *p* < 0.05 for between‐group comparison.

PTEN‐induced kinase 1 (PINK1) expression was reduced in the HF group but significantly elevated in mitochondria from the HF + LP group. Additionally, Parkin expression was markedly increased only in the HF + LP group compared to NP (Figure 4C,D). Consistent with these findings, mitophagy markers—p62, microtubule‐associated protein 1 light chain 3 (LC3II), and polyubiquitination—were elevated exclusively in the HF + LP group. Beclin, a key protein initiating autophagosome formation, was upregulated only in the HF + LP group (Figure 4C,E). Since lysosomes are essential for the fusion and degradation of autophagosomes, we assessed the co‐localization of lysosomes and mitochondria. Heart left ventricle sections showed positive co‐staining for lysosome‐associated membrane protein 2 (LAMP2) and mitochondrial cytochrome c oxidase IV (COX IV) in the HF + LP group (Figure 5A). We analyzed autophagic vacuole abundance using the gold standard of electron microscopy to further assess mitochondrial quality control (Figure 5C,D). The HF group exhibited several damaged mitochondria (Figure 5B, Panel HF, yellow arrows) and fewer autophagic vacuoles surrounding mitochondria, whereas the HF + LP group showed a significant increase (Figure 5B, Panel HF + LP, yellow arrows and Figure 5C,D).

Mitochondrial density represents the number of mitochondria relative to the size of the analyzed region. High‐fat (HF) feeding significantly reduced cardiac mitochondrial density, whereas HF combined with low protein (HF + LP) restored it to levels comparable to the normal‐protein (NP) group (Figure 5E). Similarly, mitochondrial areas were markedly reduced by HF feeding and normalized by dietary protein restriction (DPR), independent of obesity (Figure 5F). Given the changes in mitochondrial dynamics signaling, we next examined whether DPR affected network morphology. The aspect ratio, an indicator of mitochondrial elongation versus fragmentation, did not differ among groups (Figure 5G). However, circularity, which reflects the degree of mitochondrial fusion, was increased only in the HF + LP group (Figure 5H), suggesting a more fused network morphology consistent with elevated MFN1/2 expression following DPR (Figure 4A,B). These findings demonstrate that dietary DPR enhances mitochondrial dynamics and quality control, thereby further mitigating inflammaging in the heart under conditions of obesity.

### 2.4 Dietary Protein Restriction Reduces Mitochondrial Energetics in the Heart During Aging With Obesity

**FIGURE 6:**
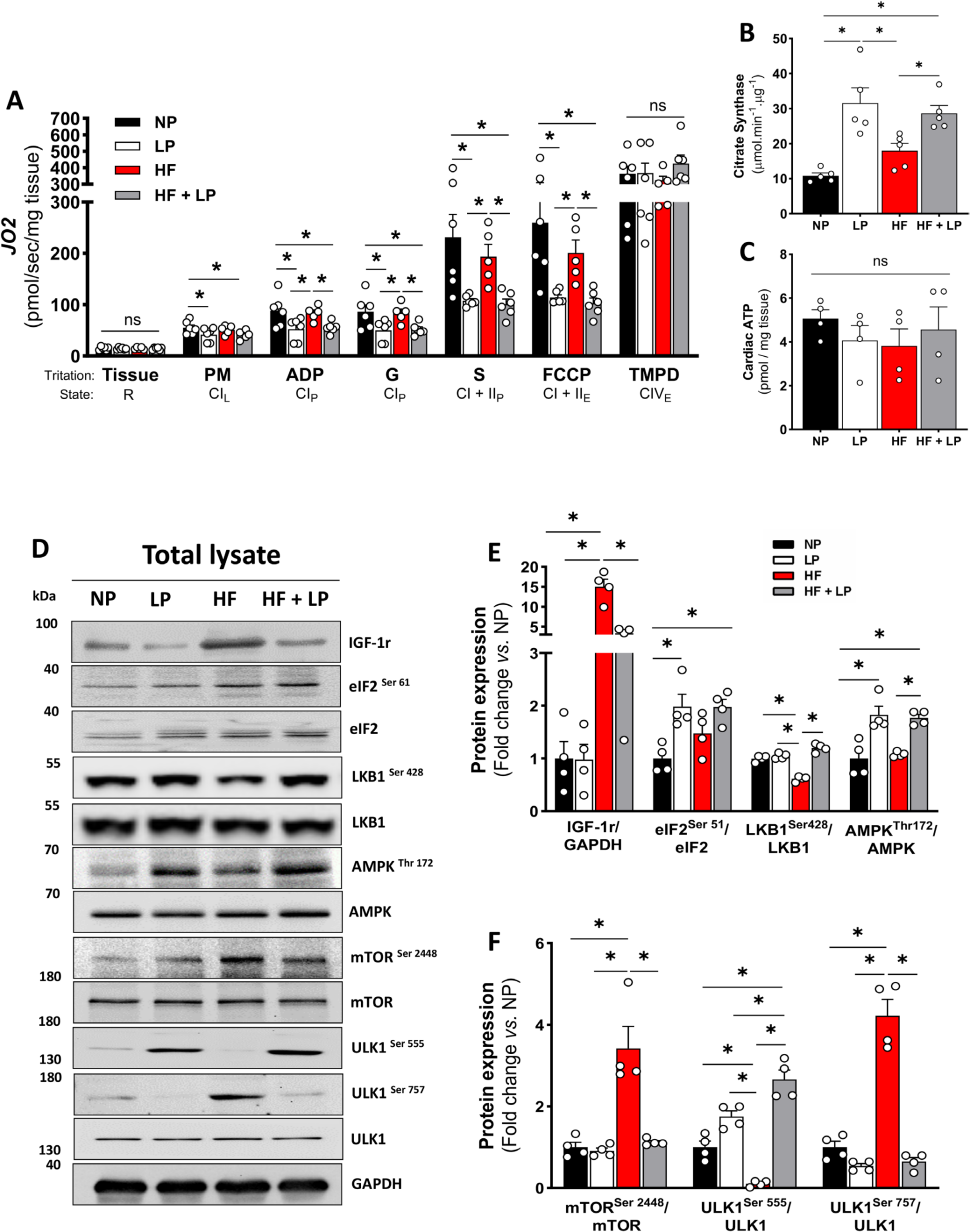
Mitochondrial respiration, citrate synthase activity, ATP levels and AMPK signaling effects on cardiac tissue following dietary protein restriction. (A) Mitochondrial respiration supported by malate, pyruvate, and glutamate (N‐linked), or succinate (S‐linked) in the presence of ADP, FCCP, and ascorbate/TMPD in heart homogenates (*n* = 5–6 per group). (B) Enzymatic activity of citrate synthase (*n* = 5 per group). (C) ATP content (*n* = 4 per group). (D–F) Representative immunoblots and densitometric quantification of IGF‐1r and GAPDH (loading control), phosphorylated and total eIF2 (Ser51), LKB1 (Ser428), AMPK (Thr172), mTOR (Ser2448), ULK1 (Ser555 and Ser757) in total heart lysates (*n* = 4 per group). Normal protein (NP), low protein (LP), high‐fat (HF), and high‐fat plus low protein (HF + LP). Data are shown as the mean ± SEM. * indicates *p* < 0.05 for between‐group comparison.

To determine whether diet‐induced changes in mitochondrial energetics mirrored improvements in dynamics and quality control during aging, we evaluated mitochondrial respiration using a substrate–uncoupler–inhibitor titration (SUIT) protocol in the heart homogenates. DPR diet reduced mitochondrial respiration in the leak oxidative phosphorylation (OXPHOS) states, irrespective of obesity (Figure 6A). While complex IV activity remained unchanged (Figure 6A), citrate synthase activity—a marker of mitochondrial content—was significantly increased with DPR (Figure 6B). Cardiac ATP content was unaffected across groups (Figure 6C). Altogether, these findings suggest that DPR remodels mitochondrial energetics by reducing respiratory capacity while maintaining ATP content by enhancing mitochondrial biogenesis, fused network morphology, and quality control, independent of obesity status.

### 2.5 Dietary Protein Restriction Restores Mitochondrial Quality Control During Aging With Obesity via AMPK–ULK1 Signaling

To investigate how DPR during obesity remodels mitochondrial energetics and enhances quality control, we next analyzed the expression of proteins involved in protein turnover and cardiac metabolism. Insulin‐like growth factor 1 (IGF‐1), a key regulator of cellular metabolism and postnatal cardiac growth during aging, is often used as a prognostic marker for cardiovascular outcomes (Abdellatif et al. 2022; Hu et al. 2024). Here, the HF diet significantly increased IGF‐1 receptor (IGF‐1r) expression, whereas the HF + LP diet significantly reduced IGF‐1r levels in the heart (Figure 6D,E). In contrast, eukaryotic translation initiation factor 2 kinase phosphorylation (eIF2^Ser61^), which plays a key role in initiating protein synthesis (Vanselow et al. 2022), was significantly increased only in response to DPR, regardless of obesity. The liver kinase B1 (LKB1) protein engages a conserved signaling pathway involving nutrient availability to autophagy initiation (Shaw et al. 2004). LKB1 is a primary upstream kinase of AMP‐activated protein kinase (AMPK) that is required to promote autophagy (Kim et al. 2011). LKB1 upregulates autophagy by inhibiting mTOR (a negative regulator of autophagy) and activating ULK1, which supports cellular breakdown of components to generate energy (Egan et al. 2011). To test whether dietary protein restriction engages the LKB1‐AMPK‐ULK signaling axis in the heart, we first assessed the LKB1 phosphorylation (LKB1^Ser428^). The HF diet significantly reduced ULK1 phosphorylation, which was restored by dietary protein restriction (Figure 6D,E). Similarly, phosphorylated AMP‐activated protein kinase (AMPK^Thr172^) was also significantly increased in response to DPR, irrespective of fat content in the diet (Figure 6D,E). Notably, the HF diet significantly increased mTOR activation, while the HF + LP diet completely abrogated this response (Figure 6D,F). Phosphorylation of ULK1 at Ser555, a site directly activated by AMPK (Egan et al. 2011) was significantly increased in response to DPR regardless of dietary fat. Conversely, phosphorylation of ULK1 at Ser757, a site activated by mTOR and known to inhibit ULK1 activity (Kim et al. 2011) was significantly increased with HF diet alone (Figure 6D,F). Taken together, these findings suggest that DPR promotes mitophagy by activating AMPK at Thr172 and increasing ULK1 Ser555 phosphorylation, while simultaneously suppressing mTOR at Ser2448 and ULK1 Ser757 phosphorylation, thereby enhancing mitochondrial quality control under metabolic stress.

**FIGURE 7:**
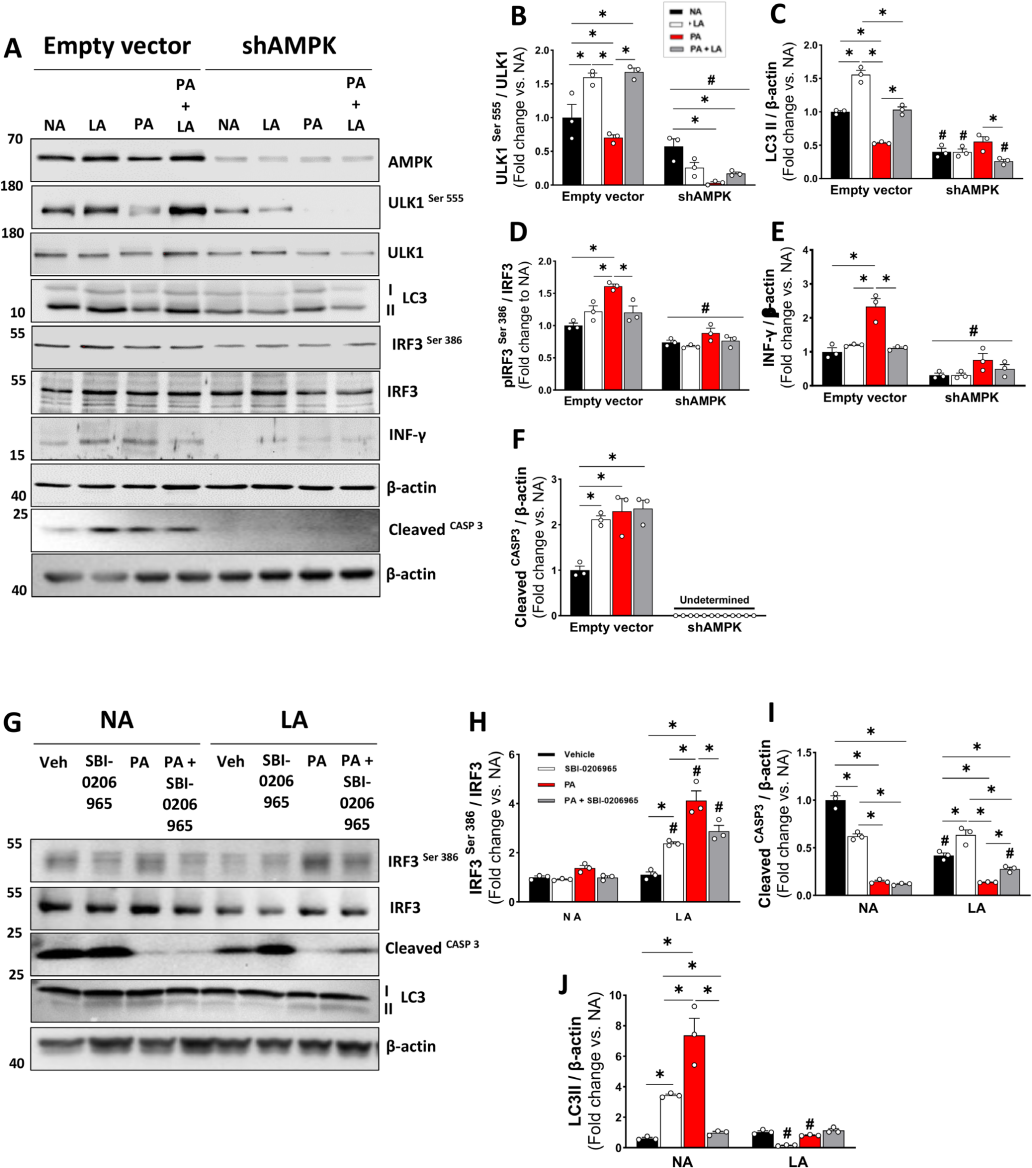
AMPK signaling is required for the mitochondrial quality control‐related signaling effects on cardiac tissue following dietary protein restriction. (A–F) Representative immunoblots and densitometric quantification of INF‐ϒ, LC3I/II, total and phosphorylated ULK1 (Ser555), total ULK1, IRF3 (Ser386), cleaved caspase‐3, and β‐actin (loading control) in cardiomyocyte lysates (*n* = 3 biological replicates per group). Normal amino acid (NA), low amino acid (LA), palmitate (PA), and palmitate plus low amino acid (PA + LA). (G–J) Representative immunoblots and densitometric quantification of phosphorylated and total IRF3 (Ser386), cleaved caspase‐3, LC3I/II, and β‐actin (loading control) in cardiomyocyte lysates (*n* = 3 biological replicates per group). Normal amino acid (NA), low amino acid (LA) under vehicle (veh), ULK1 inhibitor (SBI‐0206965), palmitate (PA), and palmitate plus ULK1 inhibitor (PA + SBI‐0206965). Data are shown as the mean ± SEM. * indicates *p* < 0.05 for within‐group comparison. # indicates *p* < 0.05 for between‐condition comparison (vs. empty vector or NA).

To determine whether AMPK directly regulates ULK1 signaling in the heart under DPR, we knocked down AMPK in H9c2 cardiomyoblast cells, which were then differentiated into cardiomyocytes and exposed to varying amino acid concentrations to simulate DPR in a cell culture environment. Using an empty vector as a control, we observed that low amino acid (LA) concentrations were sufficient to increase ULK1 Ser555 activation, regardless of the presence of palmitate (PA), which was used to mimic the HF diet in cell culture (Figure 7A,B). Using the short hairpin RNA (shRNA) approach to silence AMPK, H9c2 cardiomyocytes demonstrated a significant reduction in ULK1 Ser555 activation across all groups, with the most pronounced effect observed in the presence of PA (Figure 7A,B). Similarly, LC3II expression significantly increased in response to low amino acid (LA) conditions but was attenuated in the presence of palmitate (PA) (Figure 7A,C). AMPK knockdown reduced LC3II expression across all groups, except in the PA‐treated group. Notably, PA incubation suppressed LC3II expression in H9c2 cardiomyocytes, regardless of AMPK knockdown (Figure 7A,C). To further investigate whether DPR‐induced AMPK activation directly regulates cardiac inflammation via the cytosolic DNA‐sensing pathway, we examined IRF3 activation. PA incubation significantly increased IRF3 activation, which coincided with a marked increase in interferon‐gamma (IFN‐γ) abundance. However, PA + LA significantly reduced IRF3 phosphorylation and IFN‐γ abundance. Notably, IRF3 activation and IFN‐γ abundance were reduced considerably following AMPK knockdown, regardless of amino acid concentrations (Figure 7A,D,E). Cleaved caspase‐3, the executioner caspase of the intrinsic apoptotic pathway, was significantly increased in cells expressing the empty vector, indicating activation of regulated apoptosis (Figure 7A,F). In contrast, cleaved caspase‐3 was undetectable following AMPK knockdown, demonstrating that loss of AMPK suppresses executioner apoptotic signaling. These findings suggest that AMPK is required to maintain apoptotic competence, and its absence may impair orderly removal of damaged cells, potentially promote the persistence of dysfunctional mitochondria, and favor pro‐inflammatory stress responses.

To determine whether AMPK‐driven, DPR‐mediated mitophagy activation is ULK1‐dependent, cardiomyocytes were exposed to graded amino acid restriction in the presence or absence of the ULK1 inhibitor SBI‐0206965. Phosphorylation of IRF3 at Ser396 is a well‐established marker of innate immune activation downstream of cGAS–STING signaling. At normal amino acid (NA) concentrations, IRF3 phosphorylation remains low, indicating minimal activation of innate immune signaling. In contrast, amino acid restriction (LA) significantly increased IRF3 Ser396 phosphorylation, suggesting enhanced activation of inflammatory signaling in response to metabolic stress (Figure 7G,H). However, caspase‐3 is reduced substantially under both normal and low‐amino acid conditions when ULK1 signaling is disrupted, indicating suppression of executioner apoptotic signaling and impaired apoptotic competence (Figure 7G,I). Because LC3‐II levels were markedly increased under normal amino acid conditions (Figure 7G,J), indicating activation of autophagy/mitophagy, inhibition of ULK1 signaling prevented this increase under PA‐induced metabolic stress, demonstrating that PA‐induced autophagy is ULK1‐dependent. Notably, this autophagic response was reduced under low–amino acid conditions compared to SBI‐0206965 and PA (Figure 7G,J). The loss of this response under LA suggests that DPR may increase autophagic flux to prevent lipid‐induced autophagy activation in a ULK1‐independent manner. Collectively, these findings indicate that DPR attenuates HF diet–induced cardiac inflammaging by restoring AMPK‐mediated mitophagy through a ULK1‐dependent mechanism.

## 3 Discussion

Dietary protein restriction (DPR), an intervention that limits protein intake independent of calorie intake, has been shown to extend lifespan and improve overall health (Hill et al. 2022), suggesting that the benefits are not solely due to reduced energy consumption. Emerging evidence indicates that DPR influences mitochondrial function and quality control, which may contribute to reduced cardiovascular risk and ultimately promote longevity (Zhang, Ye, et al. 2023; Kitada et al. 2019). In this study, we demonstrate that dietary DPR mitigates obesity‐induced cardiac inflammaging by restoring mitochondrial quality control via AMPK‐ULK1 signaling. We showed that DPR reduces heart inflammaging by preventing mtDNA leakage into the cytosol, suppressing innate immune activation, and promoting mitophagy. Collectively, these findings provide mechanistic insight into how DPR exerts cardioprotective effects, highlighting its potential as a nutritional strategy to improve cardiovascular health in the context of aging and obesity.

Aging and obesity are linked to systemic chronic low‐grade inflammation and metabolic dysregulation, contributing to myocardial fibrosis, oxidative stress, and impaired cardiac energy metabolism (Owesny and Grune 2023). Adiposity, which is key to maintaining optimal body weight during aging, thereby reducing metabolic and inflammatory stress on the heart (Ren et al. 2021). In this regard, high‐protein diets have long been promoted as a strategy for weight loss, particularly in obesity and its associated metabolic complications (Gardner et al. 2007). However, higher protein intake appears to increase cardiovascular risk in both humans (Lagiou et al. 2012) and pre‐clinical models (Zhang et al. 2020). In contrast, DPR emerges as a unique dietary strategy that reduces protein intake without necessarily lowering total caloric intake, reduces fat mass, increases energy expenditure, and is associated with improvements in longevity pathways (Hill et al. 2022; Kim et al. 2025; Fontana et al. 2010). Yet, the effects of DPR on mitochondrial biology in the heart remain largely unknown.

Genomic instability may contribute to cardiovascular disease, especially under metabolic stress like aging with obesity, due to impaired DNA repair and disrupted cell cycle regulation in cardiomyocytes (Wu et al. 2024). In our study, DPR attenuates obesity‐induced cardiac remodeling during aging, as evidenced by the normalization of heart weight and the suppression of hypertrophic markers, including *Cyclin D1, NPPA, and DDB1*. The *DDB1* gene, which encodes damage‐specific DNA‐binding protein 1, is key in maintaining genomic stability via the CUL4‐DDB1 E3 ubiquitin ligase complex (Jackson and Bartek 2009). Although cardiomyocyte diameter increased in both protein‐restricted groups, this likely reflects a physiological adaptation rather than pathological hypertrophy, as there was no evidence of fibrosis. Previous studies have shown that nutrient availability influences cardiac morphology, with DPR inducing compensatory changes that enhance metabolic efficiency and stress resilience (Hennig et al. 2019).

Our transcriptomic analysis revealed that DPR downregulates the NOD‐like receptor (NLR) signaling pathway, a critical pathway in obesity‐induced inflammation. NOD‐like receptors are intracellular pattern recognition receptors that play a central role in innate immunity by detecting intracellular pathogens and damage‐associated molecular patterns (DAMPs), triggering pro‐inflammatory signaling cascades (Motta et al. 2015). Importantly, we demonstrate that DPR prevents NLR activation, indicating that protein intake influences innate immune responses in the aging heart with obesity. Toll‐like receptors (TLR) and C‐X‐C motif chemokine ligand (*CXCL*) family are the first line of immune defense responsible for recognizing DAMPs and leukocyte recruitment (Goulopoulou et al. 2016), thereby promoting inflammation (Lu et al. 2021). DPR suppressed the expression of *TLR4* and *TLR9*, key receptors that recognize mitochondrial DAMPs, further indicating that DPR limits obesity‐induced inflammaging signaling.

One of the key findings of this study is that DPR attenuates cardiac inflammaging by limiting mtDNA leakage and suppressing the cytosolic DNA‐sensing pathway. This pathway is primarily regulated by cyclic GMP‐AMP synthase (cGAS) and stimulator of interferon genes (STING) (Yu and Liu 2021), which detect cytosolic DNA and initiate the expression of inflammatory genes, contributing to cellular senescence (Yang et al. 2017). These findings align with previous reports showing that mtDNA release into the cytoplasm serves as a damage‐associated molecular pattern (DAMP), triggering sterile inflammation and contributing to age‐related cardiac dysfunction (Zanini et al. 2023; Lu et al. 2024; Grazioli and Pugin 2018). Activation of the cGAS‐STING pathway and its downstream effector, IFN‐γ, was significantly elevated during obesity but was normalized by DPR. This suggests that obesity during aging promotes chronic low‐grade inflammaging in the heart due to mitochondrial damage.

Mitochondrial dynamics is a highly conserved process involving a continuous cycle of division and fusion at both the outer and inner mitochondrial membranes (Dantas et al. 2022). Maintaining this balance is crucial for preserving the quality, integrity, and function of the mitochondrial network in the heart (García‐Peña et al. 2024). Dynamin‐1‐like protein (DRP1), a central regulator of mitochondrial fission, undergoes oligomerization through GTP hydrolysis‐driven spiral formation to facilitate membrane scission (Ji et al. 2017). Previous studies have shown that cardiac‐specific DRP1 knockout in mice with obesity completely abolished mitophagy (Tong et al. 2023). While DRP1 plays a crucial role in mitochondrial quality control during obesity by regulating multiple forms of mitophagy, the mechanisms linking this process to cardiac aging in the context of obesity remain unclear. Our findings demonstrate that DPR significantly reduces mitochondrial fission by preventing DRP1 oligomerization, a process frequently dysregulated in aging and obesity that leads to mitochondrial fragmentation (Dantas et al. 2025). This shift toward a more fused and elongated mitochondrial morphology may enhance mitochondrial efficiency and resilience to metabolic stress, thereby preserving cardiac health during aging.

We have previously demonstrated that DRP1 hyperactivation results from impaired mitochondrial quality control under conditions of aging and obesity (Dantas et al. 2022, 2025). While a severe reduction in mitochondrial bioenergetics is detrimental to cells and tissues, a mild reduction can serve important signaling roles that support cellular activities such as immune regulation (Timblin et al. 2021). This beneficial adaptation is referred to as mitohormesis, a process in which mild mitochondrial stress promotes cellular health and resilience (Cheng et al. 2023). These adaptive changes often persist beyond the initial stress exposure, resulting in long‐lasting protection (Timblin et al. 2021). The effects of mitohormesis are primarily driven by signaling pathways related to mitochondrial quality control (Ost et al. 2016), which help maintain energy balance and prevent the accumulation of dysfunctional mitochondria. In that regard, our data suggest that DPR activates mitohormesis through AMPK‐ULK1 signaling to exert mitochondrial quality control during aging, independent of obesity status.

AMPK activity is a central sensor of cellular energy status (Garcia and Shaw 2017) and can inhibit mTOR (Smiles et al. 2025), a master regulator of protein synthesis (Laplante and Sabatini 2012). Conversely, mTOR activation suppresses mitophagy, a crucial component of mitochondrial quality‐control signaling (Bartolomé et al. 2017). We then hypothesized that DPR‐induced mitohormesis occurs through AMPK‐mediated phosphorylation of ULK1, a core component of the mitophagy pre‐initiation complex (Poole et al. 2021). DPR significantly increased AMPK activation, which in turn led to enhanced phosphorylation of ULK1 at Ser555, a site known to promote mitophagy initiation (Egan et al. 2011). In contrast, ULK1 Ser757 phosphorylation, a modification driven by mTOR that inhibits ULK1 activity (Poole et al. 2021), was elevated in aging with obesity but suppressed by DPR. This reciprocal regulation of ULK1 phosphorylation suggests that DPR promotes mitophagy by activating AMPK while concurrently inhibiting mTOR, thereby facilitating mitochondrial turnover and quality control. Previous studies have established that AMPK is a key metabolic sensor that links energy status to mitophagy and mitochondrial homeostasis (Park et al. 2023). Our findings extend this paradigm by demonstrating that DPR‐induced AMPK activation is essential for maintaining quality control and limiting mitochondrial dysfunction in the aging heart with obesity. Notably, DPR increased DNAse II, an enzyme that degrades cytosolic DNA within lysosomes (Howell et al. 2003), further supporting its role in mitigating mtDNA‐driven heart inflammation. Additionally, DPR augmented autophagic vacuoles surrounding mitochondria, which, along with increased expression of mitochondrial proteins, suggests that mitophagy contributes to reducing mtDNA‐driven heart inflammaging.

To validate the functional role of AMPK in ULK1 signaling in cardiomyocytes, we performed AMPK knockdown in H9c2 cells and assessed the expression of mitophagy markers. AMPK knockdown abolished DPR‐induced ULK1 Ser555 phosphorylation and significantly reduced LC3II expression, a key marker of autophagosome formation. These findings confirm that AMPK directly regulates ULK1‐mediated mitophagy in response to DPR. Notably, DPR also inhibited palmitate‐induced IRF3 activation and IFN‐γ expression, supporting the notion that DPR attenuates obesity‐induced cardiac inflammaging through the AMPK‐ULK1‐dependent pathway. Future studies should investigate whether DPR can synergize with pharmacological AMPK activators to further enhance mitochondrial quality control and mitigate obesity‐induced cardiovascular dysfunction.

Our study exclusively examined male mice, and sex‐specific responses to dietary protein restriction (DPR) remain unexplored; therefore, it is unclear whether these findings are generalizable to females. Future studies are warranted to address these gaps and clarify the translational relevance across sexes. Although we observed robust molecular and structural cardiac adaptations, we did not assess functional outcomes, including blood markers (e.g., troponin), cardiac function (e.g., echocardiography), or exercise capacity. Nevertheless, given that previous studies have demonstrated that DPR increases lifespan, improves functional performance, and reduces adiposity in aged lean mice (Hill et al. 2022), it is reasonable to speculate that enhanced cardiac function may contribute to the observed longevity benefits following DPR. Since cardiovascular disease remains a leading cause of mortality during aging with obesity, our findings suggest that DPR possibly reduces age and obesity‐related cardiovascular risk. However, direct assessments of mortality and cardiac function are needed to confirm this possibility.

In conclusion, this study provides novel evidence that DPR attenuates age‐related and obesity‐induced cardiac inflammaging by restoring AMPK‐ULK1 signaling. By suppressing mitochondrial DNA leakage and promoting mitophagy, DPR mitigates metabolic stress and prevents the activation of inflammatory pathways in the heart. Given the challenges of adhering to long‐term caloric restriction, DPR represents a promising mitohormetic approach that maintains energy intake while conferring metabolic and cardioprotective benefits during middle age. Dietary intervention initiated in middle age serves as a prophylactic strategy to preserve overall health and protect against the onset of age‐related obesity decline in cardiovascular health. In summary, these findings highlight DPR as a potential geroprotective strategy to combat age‐ and obesity‐related cardiac dysfunction.

## 4 Methods

### 4.1 Experimental Design

All animal procedures were approved by the PBRC Institutional Animal Care and Use Committee and conducted following NIH Office of Laboratory Animal Welfare guidelines. Twelve‐month‐old male mice (Jackson Laboratory) were group‐housed (four per cage) under a 12:12‐h light–dark cycle with ad libitum access to food and water. Diets were custom‐formulated and produced by Research Diets as previously described (Hill et al. 2022), designed to be isocaloric by proportionally adjusting protein and carbohydrates while maintaining a constant fat content. The control diet (NP group) consisted of 20% casein as the protein source, whereas the low‐protein diet (LP group) contained only 5% casein. The high‐fat diet (HF group) included 60% fat and 20% casein, and the high‐fat/low‐protein diet (HF + LP group) contained 60% fat and 5% casein. All diet compositions are provided in Table S1. After 4 months of dietary intervention, at 16 months of age, animals were sacrificed during the mid‐light cycle in the fed state (unless otherwise stated) by CO_2_ exposure followed by rapid decapitation.

### 4.2 RNA Isolation

Total RNA was extracted using TRIzol reagent. Samples were homogenized in 1 mL of Trizol Reagent (Thermo Fisher Scientific) using ceramic beads in a FastPrep‐24 Tissue and Cell Homogenizer (MP Biomedicals) for 30 s at 6 m/s. Samples were left on ice for 5 min, then homogenized twice. After the final homogenization, 200 μL of chloroform (Sigma‐Aldrich) was added to each sample and mixed at 4°C for 30 min. Following a spin at 12,000 *g* for 10 min at 4°C, the RNA was collected, and 500 μL of isopropanol was added. Samples were incubated on ice for 10 min, mixed at 4°C for 30 min, and then centrifuged at 12,000 *g* for 10 min at 4°C. Then, the pellet was washed with 1 mL of 75% ethanol, vortexed, and centrifuged for 5 min at 7500 *g* at 4°C. This step was repeated at least five times. RNA concentration (ng μL^−1^) and purity (260/280) were determined using a NanoDrop 1000 Spectrophotometer (Thermo Fisher Scientific). Additionally, RNA integrity was assessed using the RNA Integrity Number (average RIN = 7.5), as determined by the Agilent 2100 Bioanalyzer (Agilent Technologies).

### 4.3 RNA‐Sequencing and Bioinformatics Analysis

RNA sequencing was conducted as described previously (Axelrod et al. 2020). Briefly, libraries were constructed and sequenced using the Lexogen QuantSeq. Briefly, library generation was performed using an oligodT primer, and double‐stranded cDNA was purified with magnetic beads. Libraries were amplified by PCR, and transcripts were sequenced in 75‐bp fragments using the NextSeq 500 (Illumina). BlueBee software was used for alignment analysis, and DESeq2 for differential expression analysis. Pathway enrichment was analyzed using ShinyGO 0.82 using the GO Biological Processes library. Differentially regulated mRNA transcripts were filtered based on the following criteria: *q* < 0.01 and base mean > 50. Quant‐Seq raw data have been deposited in the NCBI GEO database (accession GSE299132).

### 4.4 Mitochondrial Isolation

Mitochondrial isolation was slightly modified from an established protocol (Dantas et al. 2022). Fresh heart tissue was minced and homogenized in a Wheaton Glass tube with 500 μL of cold isolation buffer containing 150 mM NaCl, 50 mM HEPES (pH 7.4), and 25 μg/mL digitonin, along with phosphatase and protease inhibitors. Homogenates were incubated on ice for 10 min and then centrifuged three times at 600 *g* for 10 min. The mitochondrial supernatant was collected and washed at 7000 *g* for 10 min. Mitochondrial pellets were stored in −80°C until used.

### 4.5 Detection of mtDNA in Cytosolic Extracts

Cytosolic mtDNA content was measured as previously described (Bai et al. 2017). Briefly, the cytosolic supernatant from the mitochondrial isolation was transferred to a fresh Eppendorf tube and centrifuged at 17,000 *g* for 25 min at 4°C to obtain a cytosolic fraction free of contamination from other cellular compartments. DNA from the cytosolic fraction was isolated using the DNeasy Tissue Kit and QIAprep Spin Miniprep Columns (Qiagen). Quantitative PCR was performed on cytosolic fractions using mtDNA primers (*Dloop1‐3* and *mtND4*), nuclear DNA primers (*Tert*), and cytosolic housekeeping (*GADPH*) primers.

### 4.6 Immunoblotting

Protein extraction from heart tissue and H9c2 cells was performed as previously described (Dantas et al. 2020). In brief, cell lysates (30–60 μg protein) were resolved by sodium dodecyl sulfate‐polyacrylamide gel electrophoresis, electrotransferred to a polyvinylidene difluoride (PVDF) membrane, and incubated overnight with the appropriate primary antibody (Table S1), followed by washes and incubation with horseradish peroxidase (HRP)‐tagged secondary antibody. Signals were detected using an enhanced chemiluminescence (ECL) reagent (Thermo Fisher Scientific) and visualized with the iBright CL100 image system (Thermo Fisher Scientific). Values are expressed as fold change relative to control animals (NP group), normalized to GAPDH/β‐actin (total lysates) and VDAC (mitochondrial fraction) unless otherwise denoted. Blots were quantified using ImageJ software.

### 4.7 Quantitative Real‐Time RT‐PCR

Two micrograms (2 μg) of total RNA were converted to cDNA using a high‐capacity cDNA reverse transcription kit (Thermo Fisher Scientific) in a ProFlex PCR system (ThermoFisher Scientific). All qPCR reactions were run in 10 μL triplicate reactions containing Power SyberGreen PCR Master Mix (Thermo Fisher Scientific) using the QuantStudio 5 Real‐Time PCR system (Thermo Fisher Scientific). Primers listed in Table S2 were resuspended in nuclease‐free water according to the manufacturer's instructions (Integrated DNA Technologies). Gene expression was calculated using the 2^−ΔΔCt^ method and expressed as fold change relative to the housekeeping gene (*β‐actin*).

### 4.8 Assessment of OXPHOS and ETC Capacity in Heart Tissue

Oxidative phosphorylation (OXPHOS) and electron transfer (ET) capacity were determined by high‐resolution respirometry (Oxygraph‐2k, Oroboros) as previously described (Axelrod et al. 2020). Approximately 8–10 mg of left ventricular was collected and immediately placed into BIOPS solution (50 mM K^+^‐MES, 20 mM taurine, 0.5 mM dithiothreitol, 6.56 mM MgCl_2_, 5.77 mM ATP, 15 mM phosphocreatine, 20 mM imidazole, pH 7.1, adjusted with 5 N KOH at 4°C, 10 mM Ca‐EGTA buffer, 2.77 mM CaK_2_EGTA + 7.23 mM K_2_EGTA, and 0.1 mM free calcium). Heart homogenates were prepared as previously described (Zunica et al. 2021, 2024) to a final concentration of 1 mg/mL using additional MiR05 buffer (110 mM sucrose, 60 mM K^+^‐lactobionate, 0.5 mM EGTA, 3 mM MgCl_2_, 20 mM taurine, 10 mM KH_2_PO_4_, 20 mM HEPES adjusted to pH 7.1 with KOH at 37°C and 1 g/L de‐fatted BSA) and 2.25 mL were placed into the Oxygraph chamber. OXPHOS and ET capacity was determined using the following concentrations of substrates, uncouplers, and inhibitors: malate (2 mM), pyruvate (2.5 mM), ADP (2.5 mM), glutamate (10 mM), succinate (10 mM), tetramethyl‐p‐phenylenediamine (TMPD, 0.5 μM), ascorbate (2 mM), carbonylcyanide‐p‐trifluoromethoxyphenylhydrazone (FCCP, 0.5 μM increment), rotenone (75 nM), antimycin A (125 nM), and sodium azide (200 mM). Oxygen flux was normalized to the wet weight of total homogenized tissue (mg). Cytochrome c (10 μM) was added after the addition of glutamate to confirm mitochondrial outer membrane integrity and to ensure cytochrome c was not limiting for the measurement of each OXPHOS and ET state. O_2_ flux values were expressed relative to tissue weight per second.

### 4.9 Citrate Synthase Activity

Citrate synthase activity was determined using a commercially available colorimetric assay (Sigma). Briefly, heart lysates were centrifuged at 20,000 *g* for 15 min to pellet cell debris. The supernatant was transferred to a fresh tube, and protein content was assessed using a BCA assay (Thermo Scientific). 20 μg of protein lysate suspended in 1× assay buffer containing 30 mM acetyl‐CoA and 10 mM DTNB was plated in triplicate on a 96‐well plate. Absorbance was then measured on a plate reader set to kinetic mode (412 nm, 1.5 min duration, 10‐s intervals) before and after the addition of 10‐mM oxaloacetate. Data are expressed as μmol of activity per minute per milligram of protein.

### 4.10 Cardiac ATP Levels

Cardiac ATP concentration was determined in deproteinated heart tissue using a commercially available fluorometric assay (Abcam) according to the manufacturer's instructions. Briefly, the frozen heart (10 mg) was homogenized in 100 μL of ice‐cold 2 N PCA using 10 strokes of a handheld homogenizer, then incubated on ice for 45 min. The homogenates were centrifuged at 13,000 *g* for 2 min at 4°C. The supernatant was then transferred to a fresh tube, and the volume was brought to 500 μL with the ATP assay buffer. Excess PCA was precipitated by adding 100 μL of ice‐cold 2 M KOH, vortexing briefly, and maintaining a neutral pH. The samples were centrifuged at 13,000 *g* for 15 min at 4°C, and the supernatant was collected for ATP measurement. Standards and samples were plated in duplicate into a 96‐well black‐walled plate, the ATP reaction mix was added, and the plate was incubated at room temperature for 30 min, protected from light. The reactions were analyzed with a microplate reader (Ex/Em = 535/587 nm).

### 4.11 Immunofluorescence

The left ventricle of the heart was fixed in 10% neutral buffered formalin, embedded in paraffin, and sectioned (5 μm thick) with a cryostat. Sections were deparaffinized, rehydrated, and treated with citrate buffer for antigen retrieval before staining for mitochondrial cytochrome *c* oxidase IV (COX IV), 4′, 6‐diaminido‐2‐phenylindole (DAPI), and lysosome‐associated membrane protein 2 (LAMP2). Sections were also incubated with PBS as a negative control for staining. The images were captured with a confocal microscope (Zeiss Axiovert 220 M META confocal).

### 4.12 Transmission Electron Microscopy (TEM)

Left ventricular sections were collected at the time of necropsy and assessed for mitochondrial ultrastructure and content as described previously (Dantas et al. 2025). Briefly, small pieces of tissue were fixed by immersion in quarter‐strength Karnovsky's fixative solution. The specimens were thoroughly rinsed in 0.1 M phosphate buffer, pH 7.4, and then postfixed for 2 h in an unbuffered 1:1 mixture of 2% osmium tetroxide and 3% potassium ferricyanide. After rinsing with distilled water, the specimens were soaked overnight in an acidified 0.25% uranyl acetate solution. After another rinse in distilled water, they were dehydrated in ascending ethanol concentrations, then passed through propylene oxide, and embedded in Embed‐812 resin mixture (Electron Microscopy Sciences). Thin sections were sequentially stained with acidified uranyl acetate, followed by a modification of Sato's triple lead stain, and examined in a FEI Tecnai Spirit (T12) with a Gatan US4000 4kx4k CCD.

Mitochondrial density, area, aspect ratio, and circularity were determined by manually tracing the clearly discernible mitochondrial outlines on transmission electron micrographs and quantified using threshold‐based analysis in ImageJ. Autophagic vacuoles describe the physical features of autophagosomes, which have been counted and identified using previously established methods (Dantas et al. 2025) with the total number of autophagosomes normalized per field of view (6–10 micrograph fields per mouse).

### 4.13 Cell Culture

H9c2 cells were maintained in Dulbecco's modified Eagle's medium, supplemented with 10% (v/v) fetal bovine serum (FBS) and 1% (v/v) penicillin/streptomycin at 37°C in 5% CO_2_ in 95% air. Sub‐confluent H9c2 myoblasts were transfected with either an empty vector or short hairpin RNA (shRNA) targeting the catalytic subunit of AMPK using TransIT Transfection Reagent (Mirus Bio LLC, Madison, WI), according to the manufacturer's instructions. Stable transfectants were selected by culturing cells in medium containing 20 μg/mL puromycin. Transfection efficiency and AMPK knockdown were confirmed by immunoblotting. Given that H9c2 myoblasts' metabolism relies on glycolysis, and cardiomyocytes maintain a predominantly mitochondrial oxidative metabolism (Taegtmeyer 1994), cells were differentiated by being cultured in a low serum medium (1% FBS) in the presence of retinoic acid (10 nM; Sigma) for 10 days.

To mimic dietary protein restriction in vitro, differentiated cardiomyocytes were cultured in a modified medium that reduced amino acid availability while maintaining cell viability. Protein restriction was implemented by adjusting the concentrations of essential and nonessential amino acids in high‐glucose DMEM lacking amino acids. A custom amino acid mixture was adapted from a previously published protocol (Johnson et al. 2014), with the total amino acid concentration reduced by approximately 70% in the low amino acid (LA) group relative to the normal amino acid (NA) group. The media were replaced every 24 h, and cells were maintained under protein‐restricted conditions for 72 h. Glucose (25 mM), vitamins, sodium pyruvate, and inorganic salts were supplied at standard concentrations.

To mimic lipotoxic stress as seen in the HF group, palmitate (100 mM) was first dissolved in ethanol and conjugated to fatty acid–free bovine serum albumin (BSA) by incubating the mixture at 60°C for 10 min with constant agitation. The final BSA:palmitate molar ratio was adjusted as needed, typically to 5:1. The conjugated solution was sterile‐filtered through a 0.22 μm filter prior to use. Cells were exposed to the palmitate‐BSA solution for 4 h (Nieuwoudt et al. 2017). The ULK1 inhibitor SBI‐0206965 (25 μm, Selleckchem) was incubated overnight. After incubation, the media was aspirated, and the cells were washed twice with ice‐cold PBS before the addition of a cell lysis buffer containing 5% protease inhibitor cocktail, 5% PhosSTOP, 1% sodium orthovanadate, and 0.35% PMSF. Plates were stored at −80°C until further processing for immunoblotting.

### 4.14 Statistical Analysis

Prism 10 software (GraphPad) was used for statistical testing. Unless otherwise stated, all data are expressed as means ± standard error of the mean (SEM). Outliers were identified and removed from the dataset using Grubbs' test to ensure the validity and robustness of the results. For in vivo data, a one‐way ANOVA test was performed. For in vitro data, a two‐way ANOVA with a Bonferroni post hoc test was used to compare quantitative variables. All in vitro experiments were performed in at least three biological replicates, and the significance level was set at *p ≤* 0.05.

## Author Contributions

Wagner S. Dantas: conceptualization, methodology, validation, formal analysis, investigation, data curation, writing – original draft, writing – reviewing and editing, visualization, project administration; Elizabeth R. M. Zunica: investigation, data curation, writing – review and editing; Elizabeth C. Heintz: investigation, data curation, writing – review and editing; Charles L. Hoppel: methodology, validation, formal analysis, investigation, writing – review and editing; Cristal M. Hill: conceptualization, methodology, investigation, data curation, project administration; Christopher D. Morrison: conceptualization, methodology, investigation, data curation, project administration, funding acquisition; Christopher L. Axelrod: validation, formal analysis, investigation, data curation, writing – reviewing and editing; supervision, project administration; Gangarao Davuluri: conceptualization, methodology, investigation, data curation, writing – reviewing and editing; John P. Kirwan: conceptualization, investigation, writing – review and editing, supervision, project administration, funding acquisition.

## Funding

This work was supported by the National Institutes of Health (NIH) R01DK105032, R01DK121370 (CDM), R01DK123083 (CDM), and S10OD023703 (CDM), F32DK115137 (CMH), and K99AG070273 (CMH), U54GM104940 to J.P.K., and UL1 RR024989 (Cleveland, OH). This project used core facilities at Pennington Biomedical Research Center, which are supported, in part, by NIH center awards P30GM118430, P20GM135002, and P30DK072476. W.S.D. was supported by K99AG083239‐01.

## Conflicts of Interest

The authors declare no conflicts of interest.

## Supporting information


**Table S1:** Composition of study diets.
**Table S2:** List of reagents and resources used in the study.

## Data Availability

The data that support the findings of this study are available from the corresponding author upon reasonable request.
