# Supplementary material for: Dietary Protein Restriction Ameliorates Cardiac Inflammaging via AMPK‐ULK1‐Mediated Mitochondrial Quality Control
Source: Aging Cell. 2026 Jan 18;25(2):e70386. doi: 10.1111/acel.70386 (PMC12813272; doi:10.1111/acel.70386)
Supplement: Supplementary file 1 — Table S1: Composition of study diets. Table S2: List of reagents and resources used in the study. [file ACEL-25-e70386-s001.docx]

**Supplementary Information**

Dietary Protein Restriction Ameliorates Cardiac Inflammaging via AMPK-ULK1-Mediated Mitochondrial Quality Control

Wagner S. Dantas, Elizabeth R. M. Zunica, Elizabeth Heintz, Charles L. Hoppel, Cristal M. Hill, Christopher D. Morrison, Christopher L. Axelrod, Gangarao Davuluri, John P. Kirwan

**Appendix**

**Supplementary Table 1**. Composition of study diets.

**Supplementary Table 2.** List of reagents and resources used in the study.

**Supplementary Table 1**. Composition of study diets.

| **Research Diets Cat No: Ingredient(g)** | **D11092301 5% Casein** | **D11051801 20% Casein** | **D11092308 HF-5%Casein** | **D11092309 HF-20%Casein** |
| --- | --- | --- | --- | --- |
| Casein | 50 | 200 | 50 | 200 |
| L-Cystine | 0.75 | 3 | 0.75 | 3 |
| Corn Starch | 485 | 375.7 | 134.1 | 0 |
| Maltodextrin 10 | 150 | 125 | 125 | 125 |
| Sucrose | 107.1 | 107.1 | 107.1 | 107.1 |
| Cellulose | 50 | 50 | 50 | 50 |
| Soybean Oil | 25 | 25 | 25 | 25 |
| Lard | 75 | 75 | 242 | 242 |
| Mineral Mix S10022C | 3.5 | 3.5 | 3.5 | 3.5 |
| Calcium Carbonate | 8.7 | 12.5 | 8.7 | 12.495 |
| Calcium Phosphate Dibasic | 5.3 | 0 | 5.3 | 0 |
| Potassium Citrate | 2.4773 | 2.5 | 2.4773 | 2.4773 |
| Potassium Phosphate | 6.86 | 6.86 | 6.86 | 6.86 |
| Sodium Chloride | 2.59 | 2.59 | 2.59 | 2.59 |
| Vitamin Mix V10037 | 10 | 10 | 10 | 10 |
| Choline Bitrartrate | 2.5 | 2.5 | 2.5 | 2.5 |
| FD&C Yellow Die #5 | 0 | 0.05 | 0 | 0.025 |
| FD&C Red Dye #40 | 0.05 | 0 | 0.025 | 0 |
| FD&C Blue Dye #1 | 0 | 0 | 0.025 | 0.025 |
| **Total** | 984.8 | 1001.3 | 775.9 | 792.6 |
|  |  |  |  |  |
|  | **D11092301** | **D11051801** | **D11092308** | **D11092309** |
| **Ingredient (%)** | **5% Casein** | **20% Casein** | **HF-5%Casein** | **HF-20%Casein** |
| **gm%** |  |  |  |  |
| Protein | 5 | 18 | 6 | 23 |
| Carbohydrate | 76 | 62 | 48 | 31 |
| Fat | 10 | 10 | 34 | 34 |
| **kcal%** |  |  |  |  |
| Protein | 4 | 18 | 4 | 18 |
| Carbohydrate | 74 | 60 | 37 | 24 |
| Fat | 22 | 22 | 59 | 59 |

**Supplementary Table 2.** List of reagents and resources used in the study.

| **REAGENT or RESOURCE** | **SOURCE** | **IDENTIFIER** |
| --- | --- | --- |
| ***Antibodies*** | | |
| cGAS | Cell Signaling (1:1000) | Cat#31659 |
| STING | Proteintech (1:1000) | Cat#19851-1-AP |
| pTBK1 Ser 172 | Cell Signaling (1:1000) | Cat#5483 |
| TBK1 | Proteintech (1:1000) | Cat#28397-1-AP |
| pIRF3 Ser 386 | Cell Signaling (1:1000) | Cat#37829 |
| IRF3 | Proteintech (1:1000) | Cat#11312-1-AP |
| DNAse II | Proteintech (1:1000) | Cat#15934-1-AP |
| pJNK Thr 183/Tyr 185 | Cell Signaling (1:1000) | Cat#9255 |
| JNK | Proteintech (1:1000) | Cat#66210-1-AP |
| pNFκβ Ser 536 | Cell Signaling (1:500) | Cat#3033 |
| NFκβ | Cell Signaling (1:1000) | Cat#8242 |
| pERK1/2 Thr 202/Tyr 204 | Cell Signaling (1:1000) | Cat#4695 |
| ERK 1/2 | Proteintech (1:2000) | Cat#11257-1-AP |
| IGF-1r | Cell Signaling (1:1000) | Cat#9750 |
| peiF2 Ser 51 | Cell Signaling (1:1000) | Cat#3398 |
| eiF2 | Cell Signaling (1:1000) | Cat#5324 |
| pAMPK Thr 172 | Cell Signaling (1:1000) | Cat#2535 |
| AMPK | Cell Signaling (1:1000) | Cat#2532 |
| pmTOR Ser 2448 | Millipore (1:750) | Cat#09-213 |
| mTOR | Cell Signaling (1:1000) | Cat#2983 |
| pULK1 Ser 555 | Cell Signaling (1:1000) | Cat#5869 |
| pULK1 Ser 757 | Cell Signaling (1:1000) | Cat#6888 |
| ULK1 | Cell Signaling (1:1000) | Cat#6439 |
| Beclin | Proteintech (1:1000) | Cat#11306-1-AP |
| p62 | Proteintech (1:1000) | Cat#18420-1-AP |
| LC3I/II | Novus Biologicals (1:1000) | Cat#NB100-2220SS |
| DRP1 | Cell Signaling (1:1000) | Cat#8570 |
| PINK1 | Abcam (1:1000) | Cat#ab23707 |
| Polyubiquination | Cell Signaling (1:1000) | Cat#58395 |
| MFN1 | Cell Signaling (1:1000) | Cat#14739 |
| MFN2 | Cell Signaling (1:1000) | Cat#9482 |
| OPA1 | Proteintech (1:1000) | Cat#27733-1-AP |
| TFAM | Cell Signaling (1:1000) | Cat#8076 |
| GAPDH | Proteintech (1:2000) | Cat#10494-1-AP |
| VDAC | Proteintech (1:2000) | Cat#55259-1-AP |
| LAMP2 | ThermoFisher Scientific | Cat# PA5-118026 |
| COX IV | Proteintech | Cat# 11242-1-AP |
| Cleaved caspase 1 | Cell Signaling (1:1000) | Cat#89332 |
| Cleaved caspase 3 | Cell Signaling (1:1000) | Cat#9661 |
| pLKB1 Ser 428 | Cell Signaling (1:1000) | Cat#3482 |
| Anti-Rabbit | Millipore Sigma | Cat#GENA9340-1mL |
| Anti-mouse | Millipore Sigma | Cat#GENA931-1mL |
| DAPI | Cell Signaling (1:2000) | Cat# 8961 |
| ***Commercial products*** | | |
| Pierce™ BCA Protein Assay | ThermoFisher Scientific | Cat# 23225 |
| ***Oligonucleotides*** | | |
| Mouse *FGF21* Forward  (TACCAAGCATACCCCATCCC);  Mouse *FGF21* Reverse  (GGATTTGAATGACCCCTGGC) | Integrated DNA Technologies | NM_020013.4 |
| Mouse *β-Klotho* Forward  (CAACCTGATGATCGCCCATG);  mouse *β-Klotho* Reverse  (GCAAACCAGGCGATCTCAAA) | Integrated DNA Technologies | NM_031180.2 |
| Mouse *NPPA* Forward  (GGAGGAGAAGATGCCGGTAG);  Mouse *NPPA* Reverse  (GAGGGCAGATCTATCGGAGG) | Integrated DNA Technologies | NM_008725.3 |
| Mouse *NPPB* Forward  (AGCCAGTCTCCAGAGCAATT);  Mouse *NPPB* Reverse  (CGGTCTATCTTGTGCCCAAAG) | Integrated DNA Technologies | NM_008726.6 |
| Mouse *Cyclin D1* Forward  (CCCTGACACCAATCTCCTCA);  Mouse *Cyclin D1* Reverse  (TCTTCTTCAAGGGCTCCAGG) | Integrated DNA Technologies | NM_001379248.1 |
| Mouse mt*Loop1* Forward  (AATCTACCATCCTCCGTGAAACC);  Mouse mt*Loop1* Reverse  (TCAGTTTAGCTACCCCCAAGTTTAA) | Integrated DNA Technologies | NC_005089.1 |
| Mouse mt*Loop2* Forward  (CCCTTCCCCATTTGGTCT);  Mouse mt*Loop2* Reverse  (TGGTTTCACGGAGGATGG) | Integrated DNA Technologies | NC_005089.1 |
| Mouse mt*Loop3* Forward  (TCCTCCGTGAAACCAACAA);  Mouse mt*Loop3* Reverse  (AGCGAGAAGAGGGGCATT) | Integrated DNA Technologies | NC_005089.1 |
| Mouse mt*ND4* Forward  (AACGGATCCACAGCCGTA);  Mouse mt*ND4* Reverse  (AGTCCTTCGGGCCATGATT) | Integrated DNA Technologies | NC_005089.1 |
| Mouse *nTERT* Forward  (CTAGCTCATGTGTCAAGACCCTCTT);  Mouse *nTERT* Reverse  (GCCAGCACGTTTCTCTCGTT) | Integrated DNA Technologies | NC_000079.7 |
| Mouse *TLR2* Forward  (GCGCTTCCTGAATTTGTCCA);  Mouse *TLR2* Reverse  (AACACAGGGAACAACGAAGC) | Integrated DNA Technologies | NM_011905.3 |
| Mouse *TLR4* Forward  (TTGCTGCCAACATCATCCAG);  Mouse *TLR4* Reverse  (TACAATTCCACCTGCTGCCT) | Integrated DNA Technologies | NM_021297.3 |
| Mouse *TLR7* Forward  (AGCAGGACCATGGAAAGTGA);  Mouse *TLR7* Reverse  (TAGATTTGGCGGCATACCCT) | Integrated DNA Technologies | NM_001290755.1 |
| Mouse *TLR9* Forward  (CTCTGACTTCGTCCACCTGT);  Mouse *TLR9* Reverse  (TCAGATTCACCAGGGAGCTG) | Integrated DNA Technologies | NM_031178.2 |
| Mouse *CXCR3* Forward  (CAACTGTGGTCGAGAAAGCC);  Mouse *CXCR3* Reverse  (AGGCCTCAGTTGTCTCAGAC) | Integrated DNA Technologies | NM_009910.3 |
| Mouse *CXCL9* Forward  (ATCTTCCTGGAGCAGTGTGG);  Mouse *CXCL9* Reverse  (GTCCGGATCTAGGCAGGTTT) | Integrated DNA Technologies | NM_008599.4 |
| Mouse *CXCL10* Forward  (CCAAGTGCTGCCGTCATTTT);  Mouse *CXCL10* Reverse  (AATGATCTCAACACGTGGGC) | Integrated DNA Technologies | NM_021274.2 |
| Mouse *IL-1β* Forward  (TCAGGCAGGCAGTATCACTC);  Mouse *IL-1β* Reverse  (AGCTCATATGGGTCCGACAG) | Integrated DNA Technologies | NM_008361.4 |
| Mouse *IL-4* Forward  (TCTCGAATGTACCAGGAGCC);  Mouse *IL-4* Reverse  (ACCTTGGAAGCCCTACAGAC) | Integrated DNA Technologies | NM_021283.2 |
| Mouse *IL-6* Forward  (CCTCTGGTCTTCTGGAGTACC);  Mouse *IL-6* Reverse  (GTCCTTAGCCACTCCTTCTGT) | Integrated DNA Technologies | NM_031168.2 |
| Mouse *IL-8* Forward  (CCATGGGTGAAGGCTACTGT);  Mouse *IL-8* Reverse  (AGCTGACTTCACTGGAGTCC) | Integrated DNA Technologies | NM_011339.2 |
| Mouse *IL-10* Forward  (GCCGGGAAGACAATAACTGC);  Mouse *IL-10* Reverse  (CTGGGGCATCACTTCTACCA) | Integrated DNA Technologies | NM_010548.2 |
| Mouse *IL-13* Forward  (TGTCTCTCCCTCTGACCCTT);  Mouse *IL-13* Reverse  (GGTCCTGTAGATGGCATTGC) | Integrated DNA Technologies | NM_008355.3 |
| Mouse *TNF-α* Forward  (GTGCCTATGTCTCAGCCTCT);  Mouse *TNF-α* Reverse  (ATCTGAGTGTGAGGGTCTGG) | Integrated DNA Technologies | NM_013693.3 |
| Mouse *INF-γ* Forward  (TGAACGCTACACACTGCATC);  Mouse *INF-γ* Reverse  (TGTCACCATCCTTTTGCCAG) | Integrated DNA Technologies | NM_008337.4 |
| Mouse *INF-α* Forward  (CTCCACCAGCAGCTCAATGA);  Mouse *INF-α* Reverse  (CTCTCAGTCTTCCCAGCACA) | Integrated DNA Technologies | NM_010502.2 |
